# Continuously Tunable Optical Modulation Using Vanadium Dioxide Huygens Metasurfaces

**DOI:** 10.1021/acsami.3c08493

**Published:** 2023-08-22

**Authors:** Isaac O. Oguntoye, Siddharth Padmanabha, Max Hinkle, Thalia Koutsougeras, Adam J. Ollanik, Matthew D. Escarra

**Affiliations:** Department of Physics and Engineering Physics, Tulane University, New Orleans, Louisiana 70118, United States

**Keywords:** active wavefront control, vanadium dioxide, phase change materials, nanofabrication, Huygens metasurfaces, amplitude modulation, phase modulation, optical modulator

## Abstract

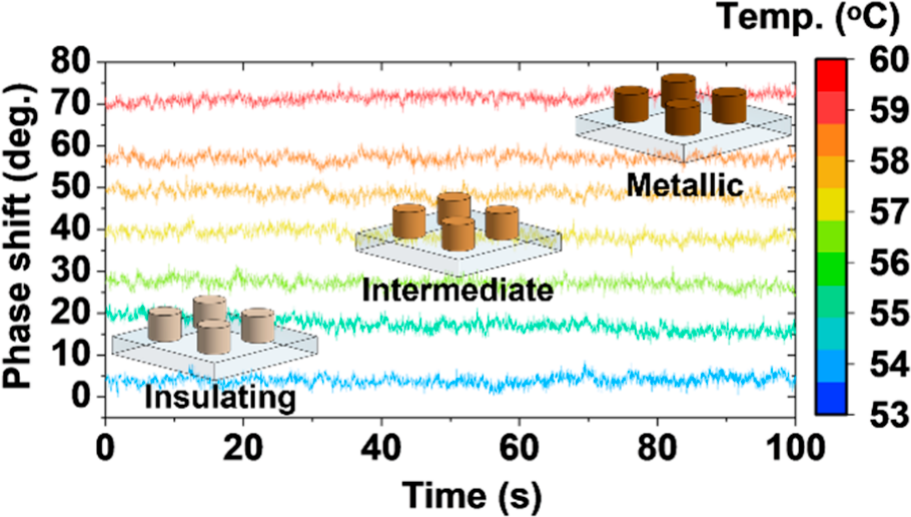

Efficient and dynamic light manipulation at small scale is highly desirable for many photonics applications. Active optical metasurfaces represent a useful way of achieving this due to their creative design potential, compact footprint, and low power consumption, paving the way toward the realization of chip-scale photonic devices with tunable optical functionality on demand. Here, we demonstrate a dynamically tunable, dual-function metasurface based on dielectric resonances in vanadium dioxide that is capable of independent active amplitude and phase control without the use of mechanical parts. Significant developments in the nanofabrication of vanadium dioxide have been shown to enable this metasurface. Gradual thermal control of the metasurface yields a computationally predicted continuously tuned amplitude modulation of 19 dB with negligible phase modulation and a continuously tuned phase modulation of 228° with negligible amplitude modulation, both at near-infrared wavelengths. Experimentally, a maximum continuously tuned amplitude modulation of 9.6 dB and phase modulation of 120° are shown, along with demonstration of stable intermediate states and repeated modulation without degradation. Reprogrammable optical functionality can thus be achieved in precisely engineered nanoantenna arrays for adaptive modulation of amplitude and phase of light for applications such as tunable holograms, lenses, and beam deflectors.

## Introduction

The need for the manipulation of light at very small dimensions is important for the design and production of next-generation integrated photonic devices. Traditional optical elements such as lenses and holograms are bulky and rely on relatively long-distance (mm to cm) variation of amplitude, phase, wavenumber, and/or polarization along the path that the light wave traverses. These bulky devices need error-free shaping and molding techniques to direct the impinging optical wave efficiently, leading to a high cost of production.^1^ Due to rapid developments in micro- and nanoscale photonics in the 21st century, there has arisen an inevitable need for the design and fabrication of small-scale optical components for easy integration in next-generation devices.

Over the past decade, research has resulted in the emergence of a diverse class of structures called optical metasurfaces. Metasurfaces are subwavelength aperiodic or periodic structures that can impart a sudden change to the amplitude, phase, polarization, and/or wavevector of optical waves at the interface between two media (over distances of nm to μm). They can be made from metallic or dielectric materials and are engineered to yield a desired optical function such as flat lenses,^2−4^ holograms,^5−7^ and biosensors.^8−10^ These metasurfaces can be designed to scatter light in the desired direction by taking advantage of their material properties as well as their geometry. Dielectric materials are preferred over their plasmonic counterparts, because of their low energy dissipation. They can also be designed to spatially confine light, resulting in electric and magnetic Mie resonance behavior which allows for full control of the optical properties of light.^11^

Although these metasurfaces have demonstrated excellent properties and performance, in some cases surpassing their traditional bulky counterparts, most of them are passive with functionality stored in the resonators that cannot be altered after fabrication. The development of active metasurfaces whose performance can be altered after fabrication is highly desirable, with far-reaching applications in varifocal lensing, dynamic holography, optical communications, and more. Dynamic tuning can be achieved through many methods, including tuning the nanoantenna array by some external stimulus, such as temperature, electric bias, or optical pumping. Other methods of tunability include tuning the nanoantenna size, spacing, and/or shape by mechanical distortion to the nanoantenna substrate or surrounding material.^12,13^ Finally, the nanoantenna material itself may be tuned by using phase change materials (PCMs) or free carrier effects.^14,15^

PCMs have particularly shown significance in paving the way for the active modulation of optical properties. They can either be nonvolatile or volatile. Nonvolatile PCMs, such as GeSbTe (GST), GeSbSeTe (GSST), and Sb_2_S_3_, undergo a binary phase change from one state to another with external stimulus. This phase change endures when the initial stimulus is removed, and the material does not undergo a reverse phase change without the input of an additional external stimulus. Nonvolatile PCMs are good candidates for memory materials, anticounterfeiting applications, and programmable photonics.^16−18^ On the other hand, volatile PCMs like vanadium dioxide (VO_2_) reversibly change their phase naturally when the initial stimulus is removed.^19^ This volatile transition enables the potential for rapid and continuous modulation of the optical properties. Vanadium dioxide atoms go through a structural rearrangement from their monoclinic structure in the insulating phase to a tetragonal rutile structure in the metallic phase at a temperature of ∼68 °C. The transition has been demonstrated to be switchable on subpicosecond time scales.^20^ If the material is inhomogeneous, as is often the case, then the transition can result in a gradual modulation from one state to the other over a range of stimuli (e.g., 65 to 70 °C). The volatile nature of VO_2_ has resulted in the use of this material for thermal regulation and tunable waveguides in the near-infrared regime. VO_2_-based integrated photonic devices have also been demonstrated for optical memory applications.^21,22^

**Figure 1:**
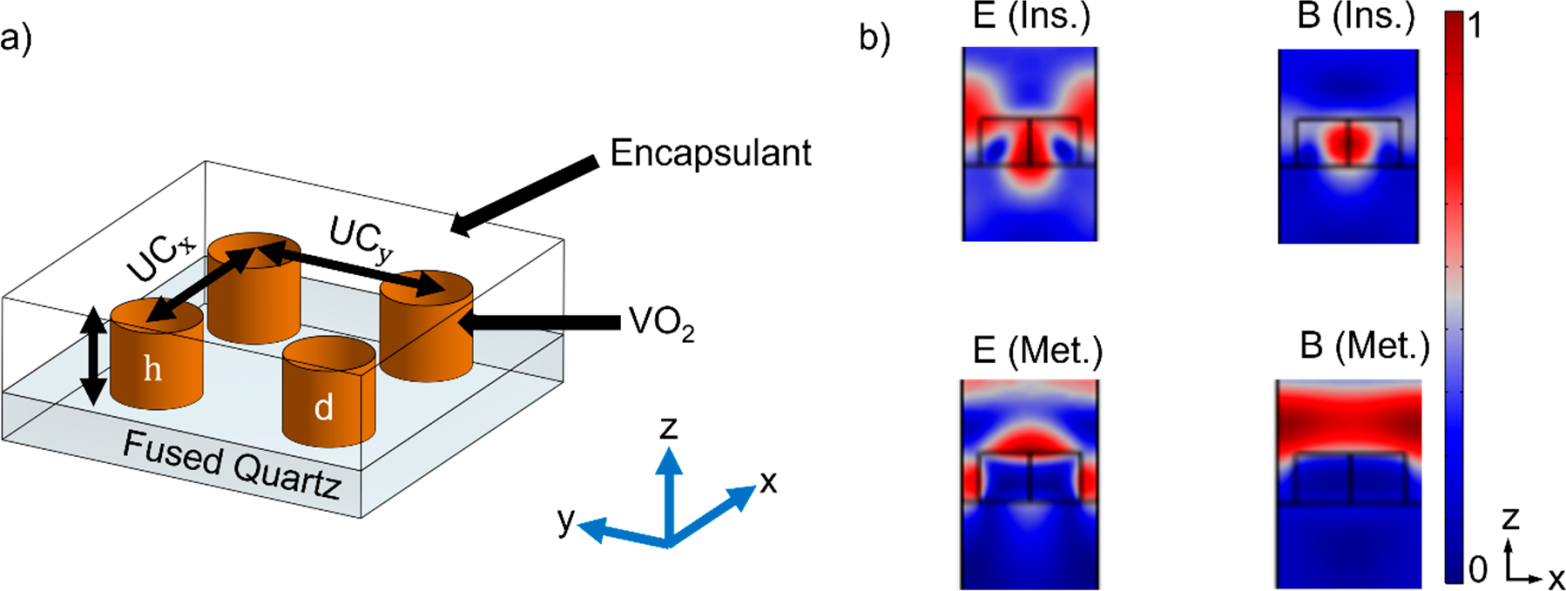
Unit cell definitions and field profiles. (a) Schematic of the Huygens nanoantenna array used in this work showing the material geometry and domains. UC_*x*_ and UC_*y*_ stand for unit cell dimensions in *x* and *y* axes, respectively. *h*_VO2_ is the height, and *d* is the diameter of the nanoantenna element. (b). Field confinement profiles of both the excited electric and magnetic fields were obtained within VO_2_ nanoresonators. These show that there is good confinement within the resonators in the insulating phase and illustrate the decay of the electric and magnetic field (initially confined within the resonator, moving to the low loss region outside the nanoantennas) in the metallic phase. The electric field and magnetic field magnitudes have arbitrary units here.

VO_2_ can also be deployed for nanoscale manipulation of the optical properties of light as a tunable thin film layer or a tunable nanostructured material. When used as a tunable layer, it provides dynamic reconfigurability of an adjacent nanostructure. Examples include tunable reflectarray modulators,^23^ tunable metasurface absorbers, and more.^24−26^ Using VO_2_ as a tunable thin film layer in a multilayer metasurface poses fabrication challenges and leads to less optical field confinement in VO_2_ relative to that in resonant VO_2_ structures, resulting in unoptimized metasurface performance. In some cases, a VO_2_ thin film layer has been incorporated to support tunable plasmonic resonances in a hybrid structure; however, the absorbing nature of the material at near-infrared wavelengths in its plasmonic state limits its optimal modulation efficiency^27^ When VO_2_ is used as the nanostructured material for the metasurface, greater optical confinement in the metasurface can be achieved via resonant interactions. For example, Bukatov et al., illustrated dual plasmonic and dielectric resonant behavior using VO_2_ thin films, wire arrays, and disk arrays at near-infrared wavelengths.^28^ Also, Kepič et al., showed optical tunability of VO_2_ nanodisks in the visible light range.^29^ Each of these groups could demonstrate some amplitude modulation of the fabricated disks but did not show a continuously tunable phase modulation. Dynamic amplitude modulation is useful for thermally switching applications such as active amplitude modulators; however, it is highly profitable to achieve incessant amplitude and phase modulation using the same metasurface design. In this work, VO_2_ is used as the nanoantenna material in Huygens metasurfaces (as shown in Figure 1a), which are known for their highly sensitive spectrally overlapping electric and magnetic dipole Mie resonances in carefully structured dielectric materials.

Although VO_2_ has a high refractive index modulation between its insulating and metallic phase, it is significantly lossy over a large range of the near-infrared spectrum (800 to 1870 nm) in its insulating phase and over a much wider wavelength range in its metallic phase. This makes it challenging to achieve good optical modulation in nanophotonic designs in that region due to its highly absorbing nature over this range. Figure 1b shows how, for the visible and near-infrared wavelength range, the well-confined electric and magnetic field in the insulating phase decays and shifts in the metallic phase due to the buildup of dissipative losses in the material. To harness the intrinsic tunability of the refractive index in VO_2_ with high performance, resonant metasurfaces may be developed in the near-zero loss region (1960 to 2500 nm and beyond) for the insulating phase. In this work, we focus on the design and experimental demonstration of Huygens metasurfaces which support spectrally overlapping electric and magnetic dipole Mie resonances, thereby enhancing forward scattering, and maximizing performance for modulation of transmitted light. The presence of overlapping dipole resonances at higher wavelengths yields higher-order resonant modes at lower wavelengths, which we also take advantage of, and allows for multiplexed optical modulation of both amplitude and phase in a single switchable nanophotonic device.

## Results and Discussion

### Survey of VO_2_ Metasurface Designs

Huygens metasurfaces supporting overlapping electric and magnetic dipole resonances can be achieved by using a broad design space. For this work, the optimal design selected should yield the largest continuous amplitude or phase modulation at individual wavelengths with little or no residual change in the other property. That is, if the amplitude (phase) is tuned continuously, then there is little or no phase (amplitude) change. Resonant nanostructures permit decoupling the modulation of individual optical properties and so outperform nonresonant thin films, whose performance is based on propagation. Resonant metasurfaces are a better choice for approaching the fundamental limit of optical performance for modulators.^30^

In pursuit of an easily fabricable VO_2_ metasurface with the best optical performance, we swept across a broad design space. These metasurfaces are designed with COMSOL Multiphysics software, which uses the finite element method for obtaining the optical performance of the design. Periodic boundary conditions are applied to the model to simulate an infinitely extended nanoantenna array in two dimensions. To simulate dynamic optical manipulation, we adopted a tuning parameter, “TuneFrac”, which represents a fractional variation in the optical properties (*n* and *k*) of VO_2_ as we tune from its insulating to metallic phase. We use temperature dependent ellipsometry on our synthesized VO_2_ thin films to extract the *n* and *k* data used for the simulations reported in this work, as shown in Supporting Information Figure S-3. Measuring our films is essential, as the optical properties of as-grown VO_2_ thin films depend on growth methods, postgrowth treatments, and defect concentration.^31^Table 1 shows schematics of the unit cell of different designs that we investigate. Each nanoantenna geometry is simulated in a periodic array using fused silica as the substrate and PDMS as the encapsulant layer, as shown in Figure 1a. The low index of the substrate and encapsulant domains yields a high refractive index contrast between the VO_2_ nanoantenna and its surroundings, which enables a high optical field confinement in the resonators. Also, selecting an encapsulant with optical properties like the substrate ensures refractive index matching, which is useful for highly transmissive optical devices. We first investigate an idealized VO_2_ metasurface design, where we have set the losses to zero. Then we observe how the absorption increases when tuning from the insulator to the metal phase. We observe resonant-enhanced absorption across all the nanoantenna element designs investigated and a resonance decay as well as a redshift as it approaches the metallic phase; the hole array is an exception, where there is a blue shift. This resonance decay is comparable to a classical damped oscillator whose damping is increased due to resonance-amplified absorption.^32^ This could prove to be significantly useful in the design of active optical switches with fast switching times on the order of milliseconds when switched thermally. To quantify the relative impact of this effect for each geometry, we show the resonant absorption after increasing the loss to *k* = 0.1. Table 1 summarizes the optimized performance of each design for both amplitude (Δ*T*) and phase (Δφ) modulation.

**Table 1:**
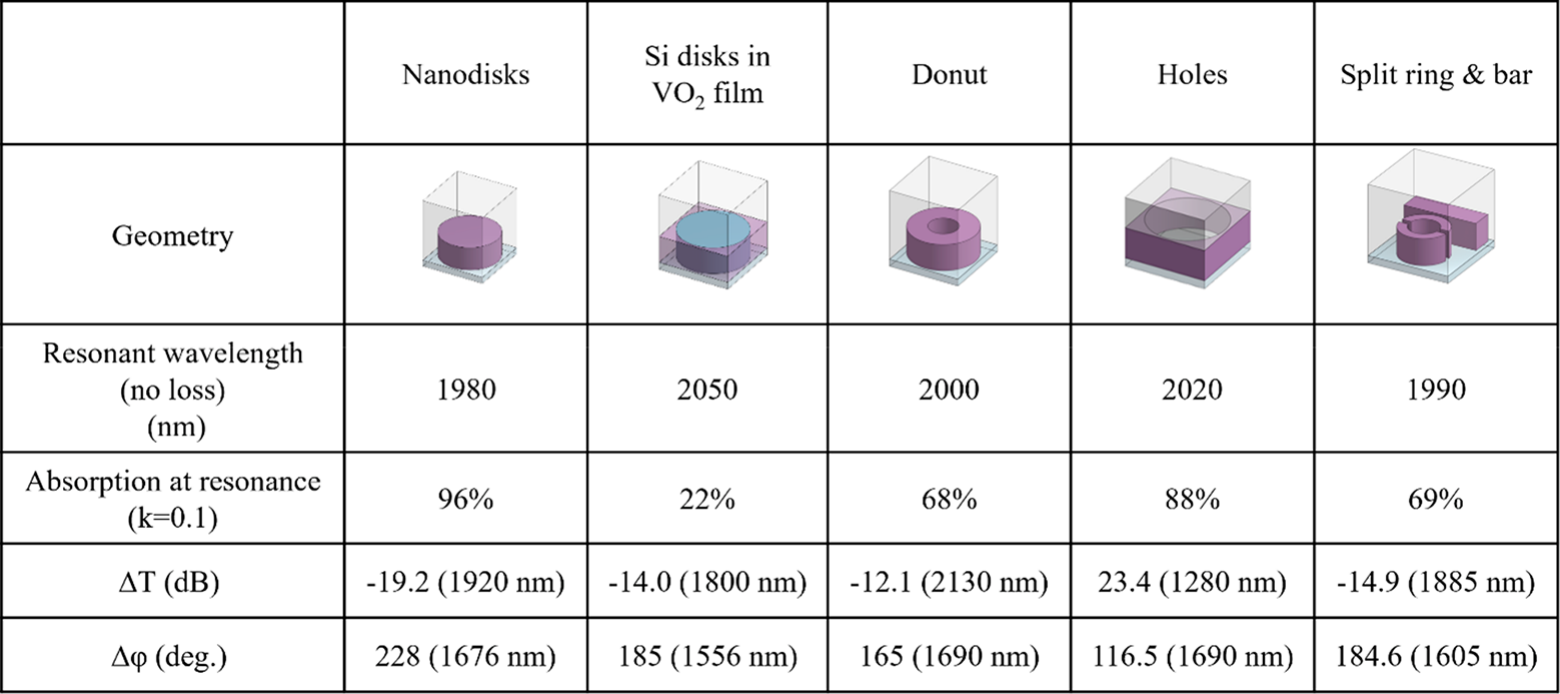
Optical Performance of Various Designs of VO_2_ Huygens Metasurfaces

The nanodisk geometry is chosen for further study as it gives the best combination of amplitude and phase modulation potential, in fact enabling us to fabricate a phase and amplitude modulator on the same chip. Other designs investigated have inclusions or voids created within them that lead to variations in spectral resonance intensity and location, thereby altering their modulating properties. The details of these design geometries are shown in Table S-1, and the simulation results are detailed in Figure S-1.

### Design of VO_2_ Nanodisk Metasurfaces

**Figure 2:**
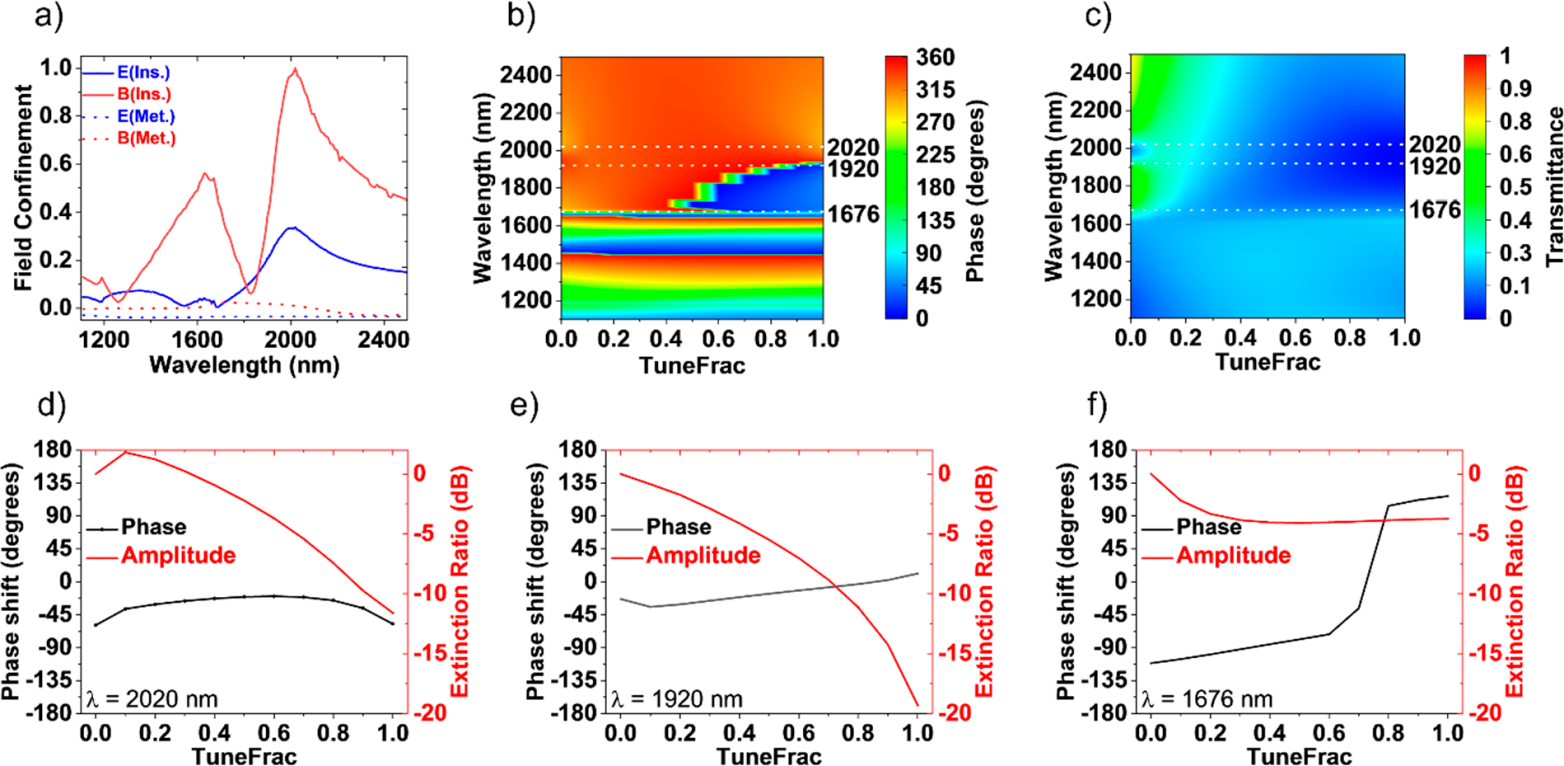
Tunable nanodisk metasurface model. (a) Normalized field confinement at the center of the VO_2_ nanodisks in the insulating (in situ) phase, showing a spectral overlay of electric and magnetic dipole modes at 2020 nm and a higher-order magnetic resonance at 1630 nm. The field confinement in the metal (Met.) phase is shown as well. (b) Modeled spectrum of continuously tunable optical phase and (c) modeled spectrum of continuously tunable transmittance shown vs TuneFrac, from the dielectric phase to the metallic phase of VO_2_. (d) Amplitude and phase modulation at the spectrally overlapping dipole mode (2020 nm). (e) Amplitude and phase modulation along the slope of the spectrally overlapping resonant modes show large amplitude change (−19 dB) with little residual optical phase change (35°). (f) Amplitude and phase modulation around the higher-order magnetic resonant mode (1676 nm) showed an optical phase tuning of 228° with little residual amplitude modulation (4 dB).

The disk geometry is carefully engineered to excite interfering magnetic and electric dipole resonances at 2020 nm. This wavelength is chosen because it lies in the low loss wavelength range for VO_2_, where spectrally overlapping electric and magnetic dipole modes can be excited and yield a useful performance. The dielectric nature of the VO_2_ nanoantennas in their insulating phase allows for the excitation of multiple Mie resonant modes, increasing the degrees of freedom available for optical modulation.^33^ We observe a strong higher-order magnetic mode at a shorter wavelength relative to the fundamental resonant wavelength of the metasurface where spectral overlap occurs; both are shown in Figure 2a. Using our tuning parameter, TuneFrac, the effects of decreasing refractive index and increasing absorption on both the amplitude and the phase of the impinging light wave in resonant and nonresonant regimes of the spectrum were investigated as shown in Figure 2b and c. TuneFrac is a custom parameter that expresses the optical constants of the intermediate mixed VO_2_ material phase (*P*_int_) as a function of (and in a range between) the optical constants of the pure dielectric (*P*_d_) and metal phases (*P*_m_) of VO_2_, as shown in eq 1 below. *P* represents the optical constant, which can be the refractive index (*n*) or extinction coefficient (*k*) of VO_2_. This enables computational modeling and analysis of the VO_2_ optical performance as a function of its continuously tunable optical properties.
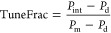
1Figure 2d shows the amplitude modulation at the resonant wavelength of the spectral overlap between the electric and magnetic dipole modes. Amplitude modulation of ∼11 dB is obtained at resonance with a negligible phase shift (∼40°). The intrinsic damping present in VO_2_ is amplified at this wavelength, precluding optimal amplitude modulation. For optimal amplitude modulation, we choose an off-resonant spectral location where we can minimize the effects of absorption enhancement as we tune from dielectric to metallic behavior. Figure 2e shows that at 1920 nm (off-resonant wavelength), we obtain a continuously tunable amplitude change of ∼19 dB with a minimal phase shift (∼35°). This photonic device can be useful in variable optical attenuators and has the potential of replacing conventional variable optical attenuators which require mechanical parts for operation.^34^ For phase modulation, we choose to operate in the vicinity (1676 nm) of the higher-order magnetic resonance, as this resonance yields a total continuously tunable phase shift of 228° (Figure 2f) with little residual amplitude shift (∼4 dB). The phase modulation without amplitude modulation obtained for this higher-order resonance mode suggests that absorption has minimal effect on this resonance. Using either stacking (transmission mode) or a mirror (reflection mode) to achieve >360° tunable phase shift, such a modulator could be engineered for use in low-loss, high-speed, and continuously reconfigurable optical wavefront shaping devices.

## Experimental Methodology

### VO_2_ Metasurface Fabrication

**Figure 3:**
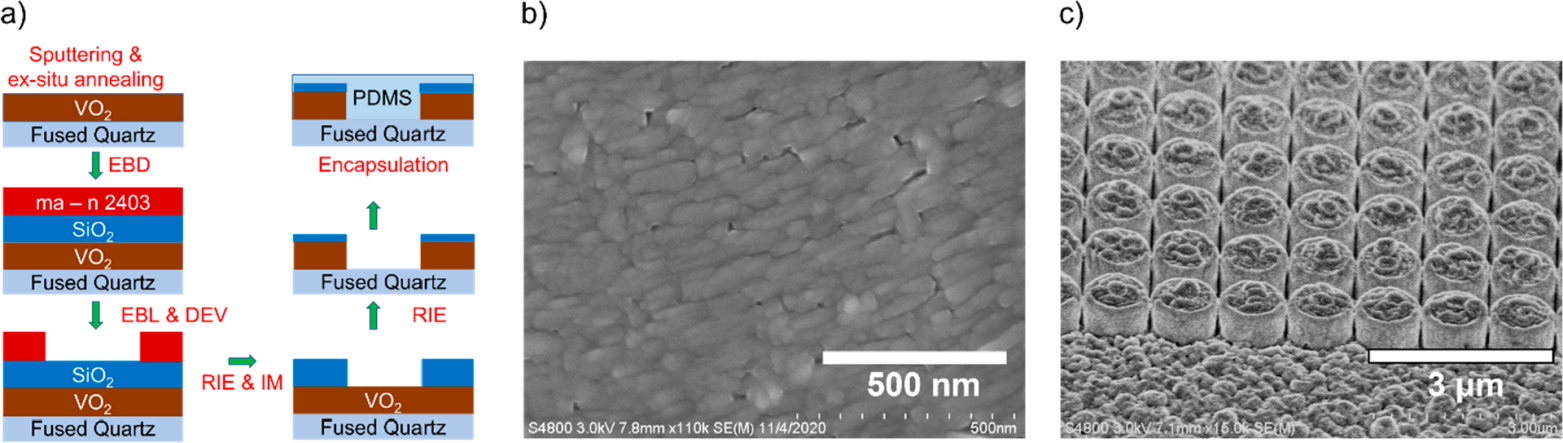
VO_2_ metasurface modulator fabrication. (a) Schematic showing the fabrication pathway for a nanostructured VO_2_ Huygens optical modulator. EBD (electron beam deposition), EBL (electron beam lithography), DEV (development), RIE (reactive ion etching), and IM (ion milling). (b). Scanning electron micrograph of ex situ annealed VO_2_ thin film. (c). Anisotropically etched VO_2_ nanodisks were fabricated using a SiO_2_ hard mask layer to protect the VO_2_ nanoantennas from being etched. This is advantageous because any leftover unetched SiO_2_ mask does not alter the modulating performance of the metasurface once it is encapsulated in PDMS (Figure S-5).

The VO_2_ thin films used in this work were made using sputtering from a VO_2_ source and ex situ annealing in a tube furnace (details in the Supporting Information Section 3). Vanadium, as a transition metal, has multiple oxidation states and form many thermodynamically stable oxides.^35^ Many of these oxides have a transition from insulating to metallic behavior; however, for most of them, the transition temperature is significantly higher than room temperature, which is less desirable for thermal or nonthermal modulation. This multiplicity of stable oxide states makes it challenging to obtain stoichiometrically accurate VO_2_. VO_2_ can also exist in different polymorphic forms which have different crystalline and electronic structures, yielding unique electrical, optical, and chemical properties due to their intense electron correlation.^36^ Different growth methods and conditions also result in different grain sizes and sample uniformity, which affects the optical properties of the material and can cause deviations over large area nanostructures. Thus, the thin film growth step requires careful optimization to obtain tunable spectroscopic optical properties that are consistent and repeatable. The selected method was chosen over several other synthesis methods that we thoroughly explored experimentally for comparison, including pulsed laser deposition, solution processing, and rapid thermal processing.^37−40^ An electron micrograph of the resultant film is shown in Figure 3b, and extensive characterization results for these films are presented in Supporting Information Figures S-2 and S-3.

The nanodisks were fabricated from their thin film precursors using electron beam evaporation of a SiO_2_ hard mask, electron beam lithography, and two steps of reactive ion etching with an ion-milling step prior to the second etch step, as shown in Figure 3a (details are summarized in the Methods section and described in detail in Supporting Information Section 5). The ion-milling step is used to remove any residual electron beam resist as well as etch deposits that form vertical walls around the SiO_2_ hard mask during the SiO_2_ reactive ion etch.^41^ Etching VO_2_ anisotropically presents a unique challenge in selecting the right etch recipe and hard mask to achieve nanostructures with smooth vertical sidewalls. Fluoride-based etch gases yield high etch rates due to the high volatility of vanadium fluorides but tend to result in isotropically etched nanostructures. Some of our experimental outcomes using fluorine-based etch recipes are presented in Figure S-4. On the other hand, chloride-based etch gases result in slow etch rates and significant redeposition due to the high boiling points of most vanadium chlorides.^42,43^ By heating the sample at an elevated temperature (100 °C) during the etch process and carefully optimizing the etch rate for the sample, well-defined vertical sidewalls can be repeatably obtained by using the chlorine-based etch recipe, as depicted in Figure 3c. This level of anisotropy in nanopatterned VO_2_ is rare in the available published literature.

### Continuous Amplitude Modulation in Thermally Tunable VO_2_ Nanoantennas

**Figure 4:**
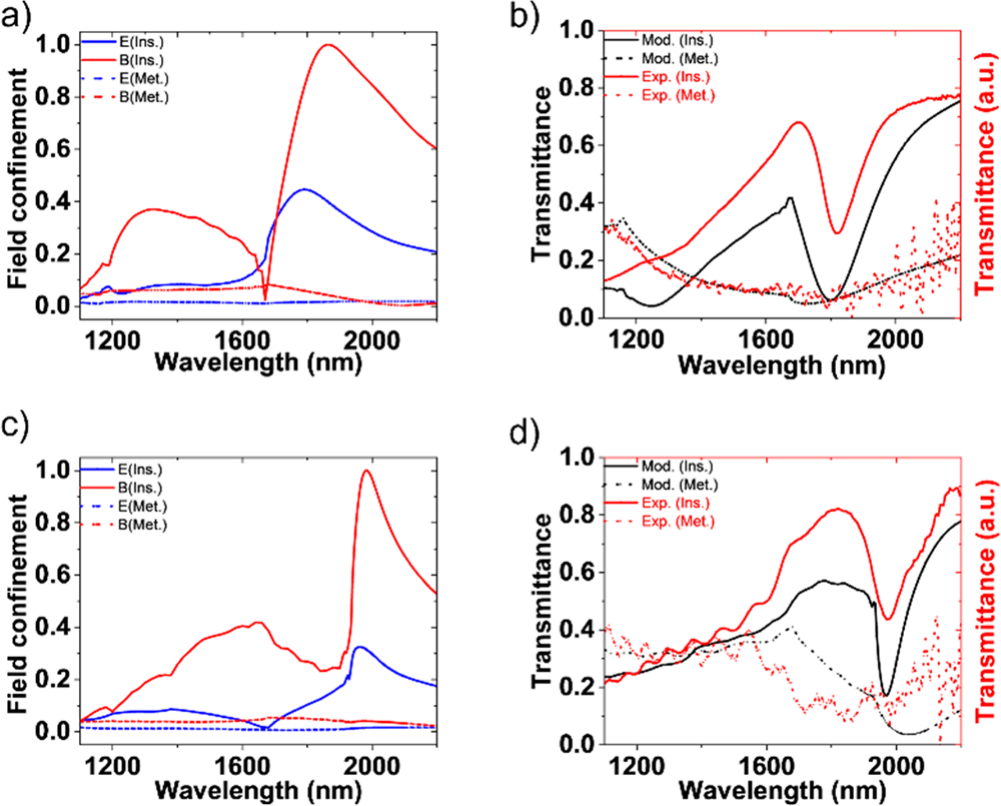
Optical performance of VO_2_ nanodisks. (a) Modeled electric and magnetic field confinement at the center of the nanodisks based on fabricated geometries before encapsulation (in air). (b) Transmittance spectra of the metasurface in insulating and metal phases before encapsulation. A resonance dip is clearly visible due to spectrally adjacent electric and magnetic dipole resonances (spectral separation of 70 nm). (c) Modeled field confinement was observed at the center of the nanodisks after encapsulation. Excited resonance modes (dipole and higher order) are red-shifted from their original spectral locations in air. The dipole mode resonance spectral separation is 20 nm. (d) Transmittance spectra of the metasurface were obtained after encapsulation. As expected, the resonance dip is red-shifted and transmittance is slightly enhanced due to a negligible index contrast between the substrate (fused quartz) and the encapsulant (PDMS).

The transmittance spectrum of the fabricated nanodisks in both material phases is obtained using a spectrometer (PerkinElmer Lambda 750 S) and compared against its expected modeled results based on feedback from fabrication; the modeled field confinement corresponding with the experimentally fabricated dimensions is shown in Figure 4a, using air as the encapsulant. To obtain a closer match between model and experimental results, we included a layer of postetch fluorocarbon residue around the resonators in the model.^44^ An energy-dispersive X-ray spectrograph is taken after the last etch step before encapsulation to validate the existence of fluorine and carbon atoms on the sample (Figure S-6). Figure 4b shows that a resonant transmission dip is observed at wavelengths close to 1800 nm in both the model and the experiment. After encapsulation, it can be seen from Figure 4c that there is a redshift in the electric and magnetic resonances due to the higher index of the encapsulant, which takes into account some migration of volatile postetch residues.^14^ The electric field dipole resonance is more sensitive to the change in index of the encapsulant and shifts more to longer wavelengths than the magnetic dipole resonance, resulting in asymmetric line shape resonances. Figure 4d shows the transmittance spectrum in both the insulating and metallic phases after encapsulation. After normalizing the intensity of the measured spectra to account for losses in the experimental setup, the modeled and experimental spectral measurements agree well for both pre- and postencapsulation VO_2_ metasurfaces. As expected, we observe a spectral redshift in the transmittance measurement after encapsulation. From these insulating and metallic phase transmission spectra, we can select the wavelength for maximum amplitude modulation.

**Figure 5:**
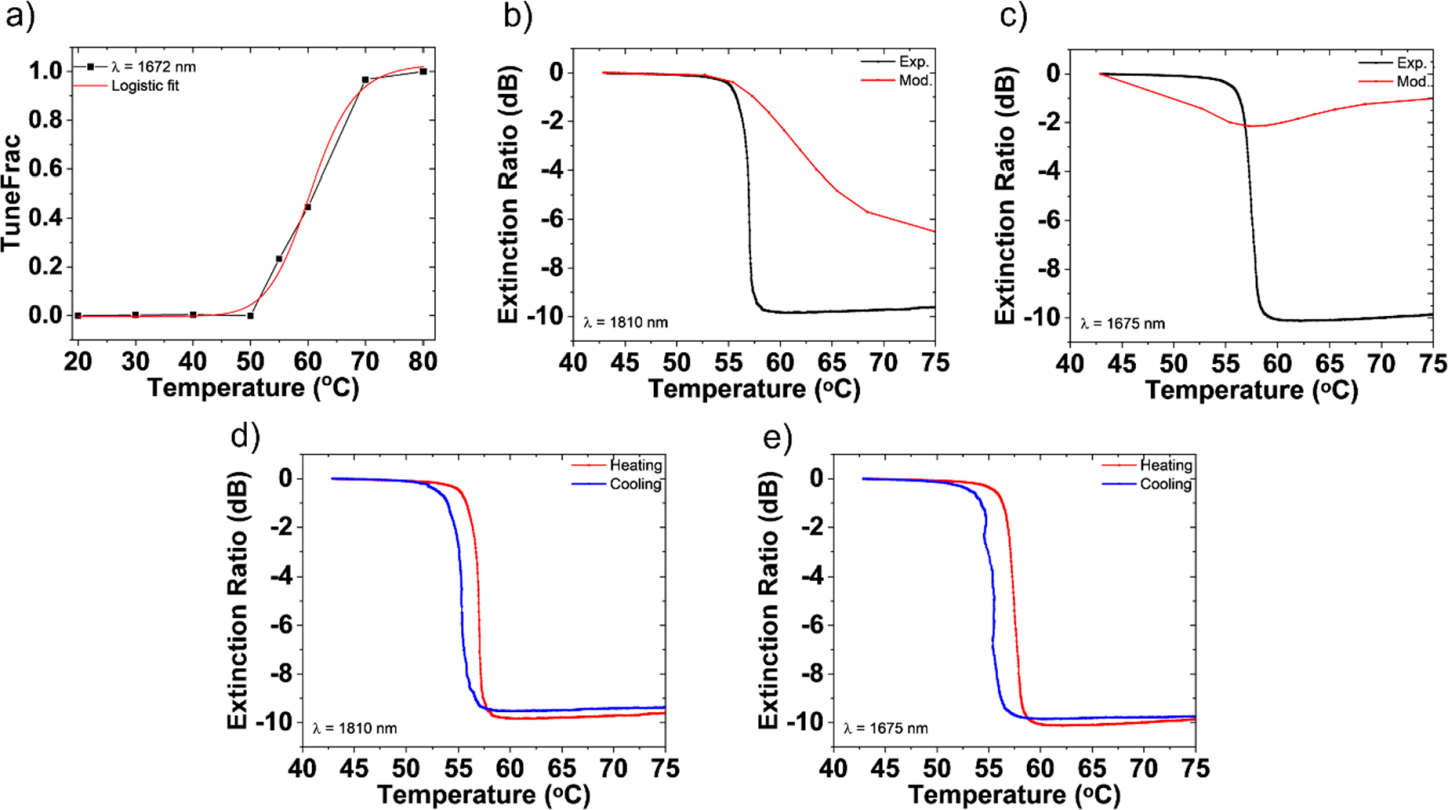
Amplitude modulation of VO_2_ nanodisks. (a) Logistic fit of TuneFrac vs temperature graph with an adjusted *R*^2^ value of 0.989. This fit was done using measured refractive index values vs temperature at 1672 nm from which each TuneFrac value was estimated as the fractional variation in refractive index from insulator to metal state. (b) Extinction ratio of the fabricated metasurface vs temperature at 1810 nm, yielding a dynamically tuned transmittance modulation of 9.6 dB between the insulating and metallic state. The model, when adjusted for the actual fabricated geometry, yields a total modulation of 6.8 dB between the insulating and metallic state. (c) Extinction ratio of the same metasurface vs temperature at 1675 nm, the wavelength at which maximum phase modulation was observed. The difference between the extinction values in modeled and measured modulation is due to fabrication imperfections along with residual redeposited etch byproducts on the sample. (d, e) Temperature-dependent hysteresis loop showing that the VO_2_ metasurface transmission returns to its initial insulating phase on cooling from its high temperature metallic phase at (d) 1810 nm and (e) 1675 nm, respectively.

To compare to the modeling results in Figure 2, a fit is established between the refractive index tuning parameter (TuneFrac) and temperature, based on the measured ellipsometric data (Figure S-3); this aids in comparing experimental and modeled amplitude modulation vs temperature at a single wavelength. Figure 5a shows that the relationship between TuneFrac and the temperature is sigmoidal. To measure amplitude modulation, the sample was mounted onto a donut-shaped ceramic heater (Thorlabs HT19R) attached to an aluminum block and vertically mounted such that laser light may pass through the heated sample in a horizontal beam path. The thermocouple was placed on top of the sample close to the location of the metasurfaces. Transmitted light was collected using an InGaAs free space amplified photodetector (Thorlabs PDA10D) aligned to the sample on the same optical axis. The impinging beam on the sample came from a supercontinuum laser (Fianium SC WL-SC-400–4) transmitted through an optical fiber with a fiber coupler at the end of the cable. The sample’s temperature is gradually tuned across the VO_2_ phase transition to obtain continuously tuned optical modulation of 9.6 dB. The model and experiment match is shown in Figure 5b for an incident wavelength of 1810 nm. The deviation from optimal amplitude modulation as depicted in Figure 2e is attributed to fabrication imperfections, which led to the relocation of each individual resonance from the originally designed wavelength. Figure 5c shows the amplitude modulation at the wavelength of the maximum phase shift (1675 nm) for this sample. The modeling result agrees well with the earlier predicted statement of minimal residual amplitude modulation; however, the experimental amplitude modulation deviates from the expected behavior. Figure S-7, the unnormalized spectral data, shows that the amplitude modulation obtained at 1675 nm is consistent with the spectral measurement after encapsulation for both the insulator and the metal states. Local variations from the modeled VO_2_ nanostructures, due to etch residue around each resonator, could lead to enhanced absorption and scattering and lower measured transmittance values in the metallic state, as the measurement setup only captures specular transmission. Improvements in fabrication, especially in a postetch clean step that is highly selective to the etch residue over the VO_2_ nanoantenna material, can help improve model and experiment correlation. Section 5 of the Supporting Information document describes other attempts made to remove postetch residue. Figure 5d and e shows the expected temperature-dependent hysteresis, revealing that the VO_2_ metasurface transitions from its monoclinic insulating phase by elevating the temperature above a heating transition temperature, stabilizes in its tetragonal metallic phase, and, on cooling to a temperature lower than the heating transition temperature, returns back to its insulating phase. This is a useful property for applications such as tunable optical memory.

### Continuous Phase Modulation in Thermally Tunable VO_2_ Nanoantennas

**Figure 6:**
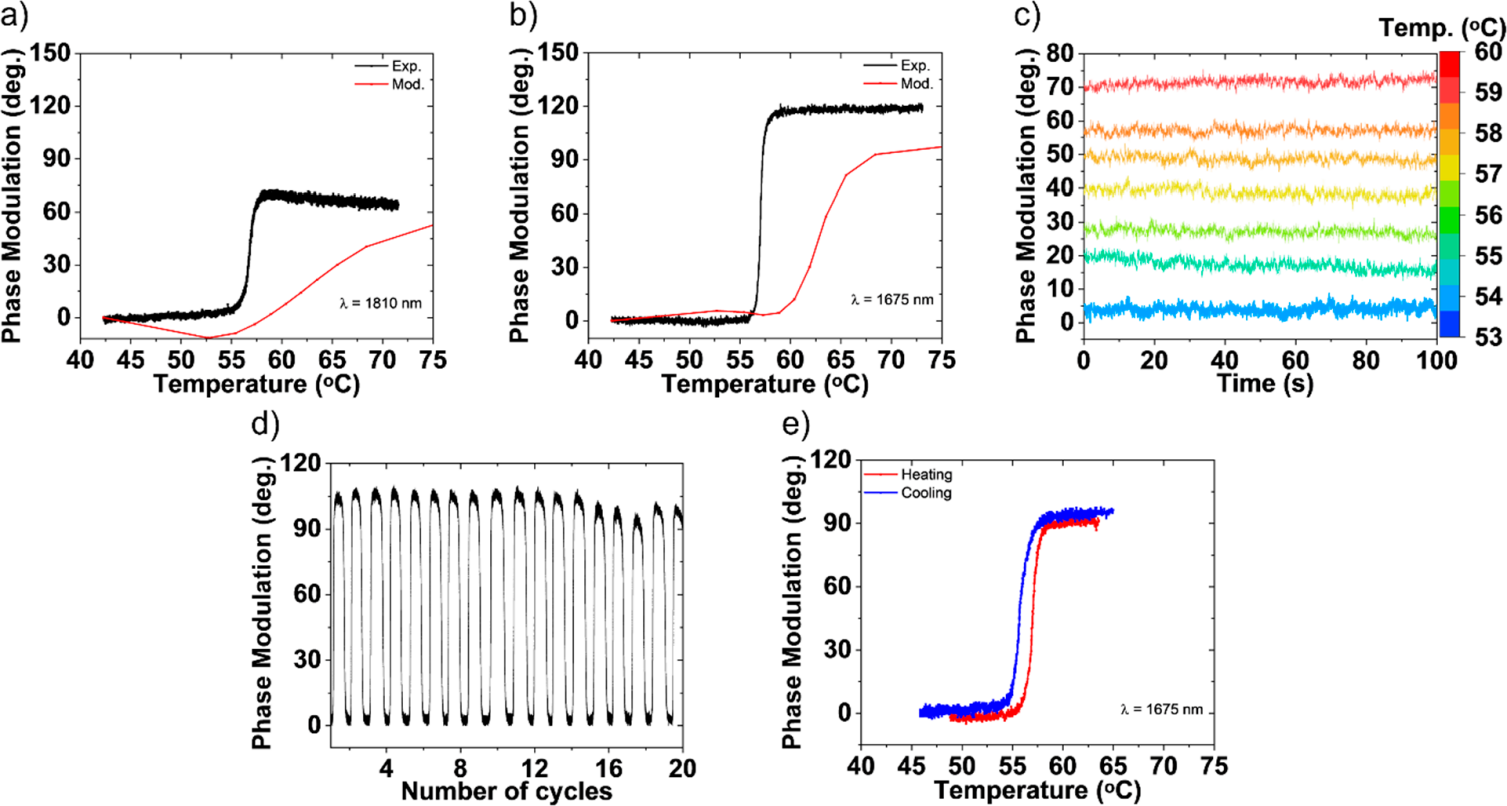
Phase modulation of VO_2_ nanodisks. (a) Transmitted phase tuning via the fabricated metasurface vs temperature at 1810 nm, resulting in 70° of continuous phase tuning. The modeled metasurface based on the fabricated geometry demonstrated a continuous phase shift of ∼53°. (b) Transmitted phase tuning via the fabricated metasurface vs temperature at 1675 nm yielded 120° of continuously tuned phase. At this wavelength, the model yielded a continuous phase shift of ∼100°. (c) Measured phase shift at 1656 nm, showing the ability to access stable intermediate phase shift at intermediate temperatures. (d) Repeated cycles of consistent phase shift as the nanodisks are switched from insulator to metal. (e) Temperature-dependent phase modulation was observed for both heating and cooling, showing a transition with hysteresis between insulating and metallic phases for one of the recurrent cycles.

For phase modulation, we model the fabricated metasurface’s optical response in the vicinity of the higher-order optically induced magnetic resonance and search for the highest phase modulation. The magnetic resonance is chosen because it decays less rapidly with increasing loss, allowing for gradual optical phase modulation with a reduced amplitude modulation. At 1675 nm, we demonstrate a maximum continuously tuned phase modulation of ∼120° as the metasurface structural and electronic properties change from the insulating to metallic state, as shown in Figure 6b. This agrees well with the model but is slightly higher than predicted, which could be due to local inhomogeneities across the metasurface sample. We measure the phase shift at the wavelength of maximum amplitude modulation (1810 nm, as shown in Figure 6a) and show that we have a residual phase modulation of ∼70°. This could be further minimized with an optimized fabrication approach, which would result in less etch residue on the fabricated nanostructures. These phase measurements were made using a custom three-beam Mach–Zehnder interferometer which uses simultaneous referencing on a nonmetasurface part of the sample to minimize the effects of drift and noise during the measurements.^45^

Despite the volatile nature of the insulator–metal transition in VO_2_, we were able to demonstrate sustained and stable phase shift measurements at intermediate fixed temperatures between the insulator and the metal states, as shown in Figure 6c. There is no significant change in the measured phase shift over about 2 min at each temperature. This ability to access intermediate states is useful toward making robust, dynamic optical modulators and reconfigurable photonic devices with tunable, and not just switchable, performance.^46^ In Figure 6d, we demonstrate repeatability of phase modulation over a narrow temperature range (from 53 °C to 60 °C) where active optical modulation is mostly pronounced. We see a consistent phase shift of ∼100° over 18 recurrent cycles, with no significant degradation. This portends that the fabricated metasurface platform can deliver repeatable performance over many tuning cycles, which is a critical metric for reliable reconfigurable photonics. Figure 6e shows a phase shift for both the heating and cooling components from one of the cycles shown in Figure 6d, revealing the expected hysteresis behavior for VO_2_-based photonics.

The VO_2_ metasurface tunable optical modulation reported here can be extended for use in beam steering applications, such as in LIDAR, by gradual spatial variation of refractive index modulation, utilizing intermediate states to create a phased array for beam deflection. This can be achieved by using a homogeneous metasurface array, where the excitation (e.g., thermal) gradient is maintained along the direction of the modulating phase, or by using a gradient metasurface, where nanoantenna geometry is varied to form a phased array and the structural phases can be collectively tuned by an external impulse.^47^

Although the VO_2_ metasurfaces reported here were designed for the near-infrared spectral range, the same design approach can be utilized to design nanophotonic devices in the visible range of the spectrum. By carefully engineering the thin film growth, relatively low loss VO_2_ (*k* ∼ 0.1 at 550 nm) can be synthesized.^48^ This could be useful for the design and fabrication of continuously tunable integrated nanophotonic devices for applications in the visible spectrum (e.g., lenses, augmented reality, and imaging devices).^29^

This VO_2_ metasurface platform can also be engineered for pixelated addressability, which is highly desirable for spatially reconfigurable photonics. Each nanoantenna array can be designed to encode the desired phase information and controlled independently by using microheaters. This can be useful for making tunable spatial light modulators and phase modulators, which require no mechanical movements for robust, compact performance. Such localized modulation of VO_2_ nanoantenna arrays via microheating can be achieved using other external stimuli, such as electrical or optical impulses. Optical tunability has been shown in previous work using a hybrid VO_2_-antenna platform at picosecond time scales.^49^ Electrical control has also been demonstrated using VO_2_ thin films as the tunable layer in multiple published works.^50−52^ This could be achieved using an array similar to that discussed in this paper by utilizing multiple fabrication steps, where metal fingers are placed between each nanoantenna element through electron beam lithography and metal lift-off. The fingers are alternately connected to one of two larger metallic contact pads, enabling the application of an electrical bias to the metasurface. This can cause local heating of the VO_2_ nanoantennas, thereby resulting in a material phase transition when the applied bias is gradually changed. Multiple pixels with different unit cell dimensions or different contact connections could then be fabricated on one chip, allowing for pixelated device control through one or more external electrical impulses. Further optimization in design and fabrication is required to allow for precise and efficient pixelated control of tunable nanophotonic performance with such an electrically controlled device.

## Conclusion

We have modeled and experimentally shown a continuously tunable Huygens metasurface optical modulator operable in the near-infrared region of the electromagnetic spectrum. The tunable properties are achieved by using a volatile PCM, VO_2_, whose optical properties can be modulated when subjected to an external impulse such as temperature, electric field, or optical pumping. In this work, the tunable optical modulation is achieved through thermal tuning. Maximum modeled amplitude modulation of ∼19 dB is achieved with a minimal phase modulation of (∼35°). The highest modeled phase modulation achieved is 228° with a small residual amplitude modulation of (∼4 dB). Through much novel process development, VO_2_ metasurfaces were fabricated successfully, and experimental results demonstrate a continuously tunable amplitude modulation of 9.6 dB and a continuously tunable phase modulation of 120°. Phase modulation was demonstrated for intermediate states and for repeated tuning cycles. Further development of VO_2_-based nanophotonics will enable high-speed, continuously tunable optical devices for use in a wide range of applications.

## Methods

### Metasurface modeling

Electromagnetic wave calculations for our dynamically tunable VO_2_ metasurfaces were performed using the finite element method (COMSOL Multiphysics RF Module). Each metasurface design consists of an encapsulating layer (poly(dimethylsiloxane) or PDMS), the nanoantenna material (VO_2_), and the substrate (fused quartz). Periodic boundary conditions are applied to the model to simulate an infinitely extending nanoantenna array in two dimensions. The encapsulating layer and the substrate material are chosen to have similar refractive index values across the relevant spectrum for index matching. These layers encase a Huygens source VO_2_ nanoantenna. The nanoantenna is chosen to achieve a high refractive index difference relative to its surroundings for a strong optical field confinement.

We modeled idealized lossless and real lossy VO_2_ metasurfaces. Table S-1 shows the geometric parameters for the metasurface designs explored in this work. Our simulation results show that our designed metasurface can achieve amplitude (phase) modulation with minimal change in phase (amplitude) across diverse Huygens metasurface designs, as shown in Table 1.

### Metasurface Fabrication

VO_2_ thin films were grown by RF sputtering vanadium oxide on a fused quartz substrate using a VO_2_ target, followed by ex situ annealing at 500 °C in a tube furnace. The nanodisks are patterned by electron beam lithography and etched by using inductively coupled plasma reactive ion etching (ICP RIE). The step-by-step details of the nanodisk fabrication are described in Section 5 of the Supporting Information document.

## Supporting Information Available

Table S-1 displays geometric parameters of the diverse Huygens metasurface designs discussed in the manuscript; Figure S-1 shows the resonant plots for idealized lossless and lossy VO_2_ Huygens metasurfaces indicating the enhancement of absorption at resonance for these designs; Figure S-2 contains different thin film properties of as-grown VO_2_ thin films on fused quartz substrates reported in this work; Figure S-3 shows thin film optical constants of synthesized VO_2_ thin films on fused quartz; Figure S-4 shows the scanning electron micrographs of some other etch recipes attempted toward obtaining highly anisotropic VO_2_ nanodisk arrays; Figure S-5 shows the modeled effect of a leftover SiO_2_ layer on the VO_2_ metasurface after the final etch step; Figure S-6 displays the electron dispersive spectrographs that show elemental composition of postetch products on the sample after the last etch step; Figure S-7 compares the unnormalized metasurface transmission spectra measurements with the corresponding model prediction (PDF)
