# Supplementary material for: Continuously Tunable Optical Modulation Using Vanadium Dioxide Huygens Metasurfaces
Source: ACS Appl Mater Interfaces. 2023 Aug 22;15(34):41141–50. doi: 10.1021/acsami.3c08493 (PMC10472332; doi:10.1021/acsami.3c08493)
Supplement: Supplementary file 1 — am3c08493_si_001.pdf [file am3c08493_si_001.pdf]

Supporting Information

Continuously Tunable Optical Modulation Using Vanadium Dioxide Huygens Metasurfaces

Isaac O. Oguntoye[1,§,], Siddharth Padmanabha[1], Max Hinkle[1], Thalia Koutsougeras[1], Adam J. Ollanik[1], Matthew D. Escarra[1]*

[1]Department of Physics and Engineering Physics, Tulane University, New Orleans, Louisiana 70118

[*]Email: ioguntoye@tulane.edu

This supporting information document includes details about the 1.) geometry of the different VO_2 metasurface designs shown in the manuscript 2.) resonant field confinement and absorbance plots for lossless and lossy VO_2 metasurfaces 3.) growth and characterization of VO_2 thin films grown on amorphous fused quartz substrates 4.) thin film temperature-dependent ellipsometry on VO_2 thin films grown on fused quartz, 5.) VO_2 metasurface nanofabrication and 6.) optical characterization of VO_2 nanoantenna geometries comparing model and experimental results.

Section 1: VO_2 Metasurface Dimensions

Geometry	Height (nm)	Diameter (nm)	Unit Cell (nm)
Disks	394	882	1162
Donut[a]	310	1020	1275
Silicon in VO_2 disks	235	880	1270
Split ring and bar[b]	430	845	1185
Holes	480	870	1110

[a] The donut geometry has an inner diameter of 295.8 nm.

[b] The bar in the split ring and bar geometry has a length of 600 nm and a width of 150 nm. The inner diameter of the ring is 240 nm and the distance from the center of the split ring to the center of the bar is 577.5 nm.

Table S-1: Modeled dimensions of VO_2 metasurfaces*. Geometric dimensions are for the metasurface unit cells presented in Figure 2 and Table 1.*

Section 2: Resonant plots for lossless and lossy VO_2 geometries

Due to the lossy nature of VO_2, the absorptive response of the resonators is amplified at resonance like a damped oscillator. This loss is often accompanied by a red shift of the resonances as well as resonance decay for most of the geometries investigated as listed in Table S-1. In the hole geometry, the resonance is blue-shifted as loss is introduced into the system, which could be due to Babinet's principle[1]. This section shows the impact of introducing a small loss (k=0.1) to an idealized lossless VO_2 system and shows the absorption enhancement at resonance for each of these geometries.

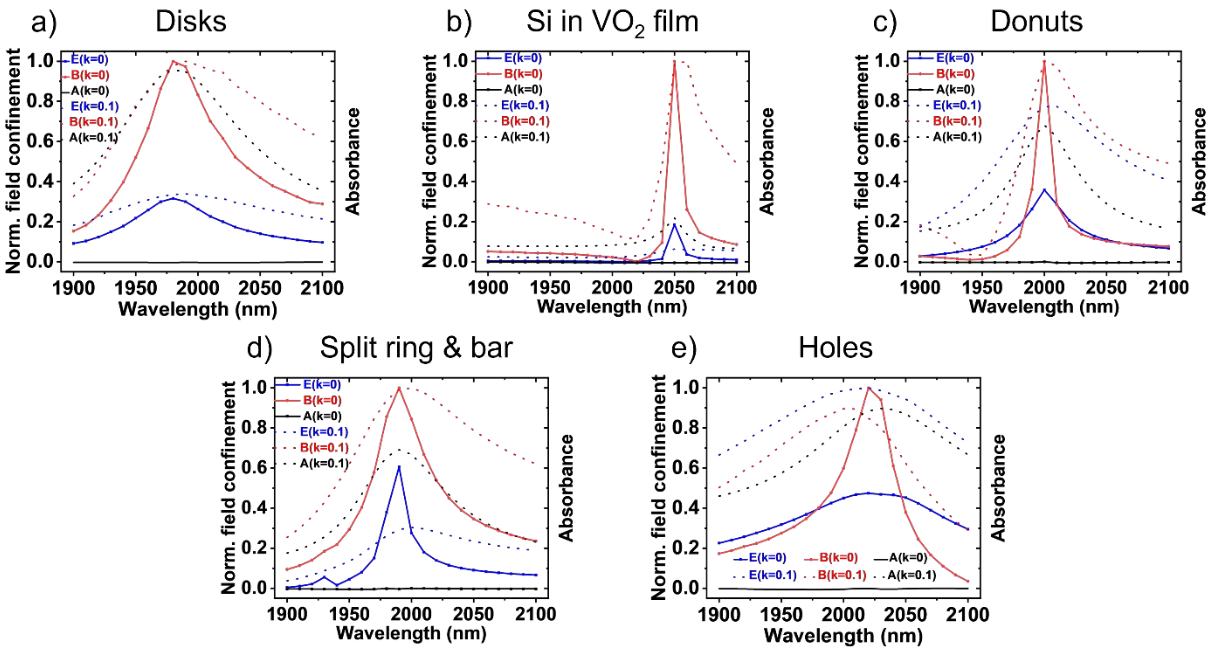

***Figure S-1**: **Resonance in lossless and lossy VO_2 metasurfaces for** (a). VO_2 nanodisks with spectrally overlapping resonances at 1980 nm, (b). Silicon disks in a VO_2 film with spectrally overlapping resonances designed at 2050 nm, (c). VO_2 donuts with spectrally overlapping resonances at 2000 nm, (d). VO_2 split rings and bars, engineered to excite spectrally overlapping resonances at 1980 nm, and (e). VO_2 holes with spectrally overlapping resonances at 2020 nm.*

Section 3: VO_2 Thin Film Growth and Characterization

VO_2 thin film growth is generally accomplished in one of two ways[2]. The first method involves a single-step synthesis of VO_2 on the substrate, where stoichiometry, uniformity, and material properties are fixed in one step. This method is achieved at high temperatures (near 500°C) to permit the crystallization of VO_2 onto the substrate. The second method utilizes a dual-step process, where a mixed-phase vanadium oxide film is vapor deposited, spun-on, or drop-casted onto the substrate. This film is then given a post-deposition treatment, yielding the desired vanadium dioxide stoichiometry with useful tunable optical properties. In this work, VO_2 thin films are grown on fused-quartz substrates using the second method by RF magnetron sputtering and

ex-situ annealing. Fused quartz substrates were chosen due to their low index and transparency at wavelengths of interest for metasurface fabrication, thus enabling a high index contrast with the VO_2 nanostructure. A VO_2 target (AJA 2" diameter) is used in the sputtering step (without substrate heating) with an oxygen-vanadium mole ratio close to the desired stoichiometry. The deposition power and pressure were kept at 200 W and 3.3 mTorr respectively for the sputtered films, using Ar plasma sputtering and no process gas. These films are then annealed in the second step in a hot wall 1" quartz tube furnace under vacuum to yield polycrystalline VO_2 films. Multiple characterization approaches are used to investigate the properties of the synthesized thin films. These include temperature-dependent transmittance, X-ray photoelectron spectroscopy, X-ray diffractometry, atomic force microscopy, and variable angle spectroscopic ellipsometry, as shown in Figure S-2. The films are confirmed to be VO_2, with high uniformity and relatively low surface roughness.

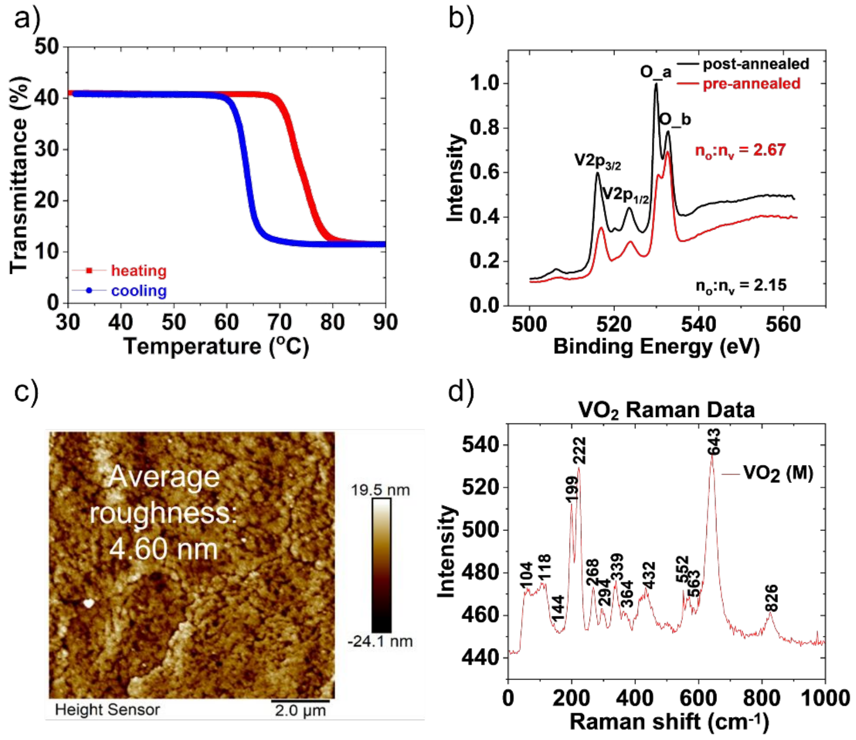

Figure S-2: VO_2 thin film characterization (a). Temperature-dependent transmittance measurement of a ~170 nm thick film at 1340 nm illumination wavelength. (b). X-ray photoelectron spectroscopy showing the mole ratio of the sputtered (pre-annealed) films and the post-annealed films. (c). Atomic force microscopy image revealing roughness of the VO_2 film after annealing. (d). Raman spectroscopy of VO_2 thin film sample showing distinct Raman peaks for the VO_2 monoclinic crystal structure.

Section 4: **VO_2 thin film ellipsometry on fused quartz substrates**

Temperature-dependent variable angle spectroscopic ellipsometry was performed on the as-grown films on fused quartz substrates. This was done using a RC2 ellipsometer (J.A. Woolam) where the sample was mounted on a custom-made heating stage as shown in Figure S-3a. The thermocouple was glued to the ceramic heater using a thermal adhesive. A temperature differential between the ceramic heater and the film surface of ~10°C was accounted for in obtaining the temperature-dependent optical constants shown below. The B-spline model was used to fit the

measured ellipsometric data for obtaining the refractive index and extinction coefficients from 210 nm – 2500 nm as shown in Figures S-3b and S-3c. Ellipsometry measurements are best fit with a graded-layer model on fused quartz. The data here represents the average index between the top and bottom indexes of the graded index layer. The average index and loss variation between top and bottom graded layers, over the range of wavelengths of interest for designing optically resonant VO_2 metasurfaces, are: Δn=0.446 and Δk=0.162 (insulating state, 20°C); Δn=0.267 and Δk=0.543 (metallic state, 80°C). The raw data shown in Figures S-3b and S-3c are available on refractiveindex.info. [NOTE: THE DATA WILL BE POSTED IN THIS PUBLIC DATABASE ONCE THE MANUSCRIPT IS APPROVED FOR PUBLICATION]

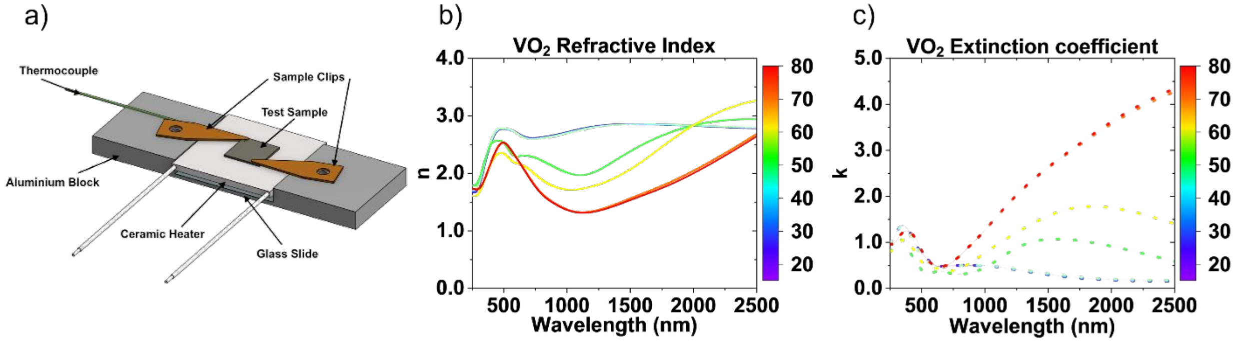

Figure S-3: VO_2 thin film ellipsometry on fused quartz substrates. *(a). Custom heating stage setup for temperature-dependent ellipsometer measurements using the RC2 Woollam ellipsometer. (b). Temperature-dependent refractive index spectra and (c). extinction coefficient spectra showing the transition from insulating to metallic VO_2, with corresponding decrease in refractive index and increase in absorption, measured using the RC2 Woollam ellipsometer.*

Section 5: VO_2 Metasurface Nanofabrication

VO_2 metasurfaces used in this work are fabricated through multiple process steps. First, thin film synthesis (discussed in Section 3 of this document) is followed by electron beam evaporation to

deposit an SiO_2 hard mask, which is useful to protect the nanostructured thin film patterns from being etched off in a subsequent step. The SiO_2 hard mask is deposited at low pressure (< 3.0 x 10^{-6} Torr) after titanium gettering to minimize flaking due to moisture-induced adsorption. Then a very thin carbon layer is deposited onto the SiO_2 surface to facilitate charge dissipation during the electron beam writing step. Two layers of an electron beam sensitive polymer (ma -n 2403) are spun onto the carbon layer, with total resist thickness of 600 nm. This is followed by development which is done in ma – D 525 developer for 1 minute and then rinsed in deionized water for 3 minutes. The sample is then taken for reactive ion etching to transfer the written patterns to the VO_2 layer. First, the unpatterned SiO_2 layer is etched off using CHF_3 (12 sccm) and Ar (38 sccm) at 50 mTorr and 1000 W ICP power with a bias power of 250 W. The electron beam resist acts as a soft mask in this process to protect the patterned nanoantennas. This step is followed by ion milling to remove the fluorocarbon layer deposited around the side walls of the nanodisks while preserving their vertical profile. The sample was inclined at 45° to the ion beam at a beam current of 120 mA and beam voltage of 600 V. The sample was etched for 4 minutes. It should be noted that significant over-etching of the nanostructures after the SiO_2 etch in the ion milling tool could lead to loss of pattern definition and worsen the optical performance of the metasurface and should be avoided. Next, the patterned VO_2 layer is etched in Cl_2 (60 sccm) and Ar (180 sccm) at 7 mTorr and 700 W ICP power with a bias power of 250 W. The substrate is heated to 100^oC during the etch to minimize redeposition and increase the VO_2 etch rate. Elemental composition of etch products on the surface of the sample are obtained using energy dispersive x-ray spectroscopy. This shows that the etch process leaves redeposited residues of fluorinated and chlorinated compounds which could in turn make the fabricated geometries differ in performance from the modeled predictions. The metasurface is then encapsulated in PDMS and is ready for full

characterization. Other etch recipes for etching VO_2 were studied and the etch outcomes are presented in Figure S-4. The electron micrographs taken show that the desired anisotropy was not achieved with these alternate VO_2 etch recipes.

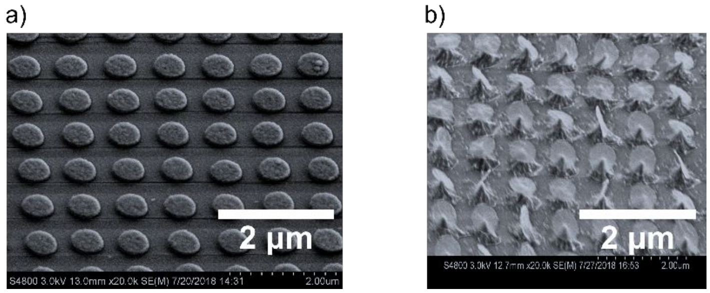

Figure S-4: VO_2 alternate etch results. *Scanning electron micrograph of VO_2 nanostructures using (a). CF_4 and O_2 etch recipe and (b). CF_4 & Ar etch recipe. Both recipes used Al_2O_3 as the hard mask.*

We modeled SiO_2 layers of different thicknesses on top of the VO_2 nanodisks to study the effects of a residual SiO_2 hard mask layer on the optical performance of the PDMS-encapsulated VO_2 nanodisks. We observe that there is no significant shift in the resonant wavelength nor change in the resonant intensity of the electric and magnetic dipole modes confined in the nanodisks due to this residual SiO_2 layer that may remain on top once the nanofabrication is complete.

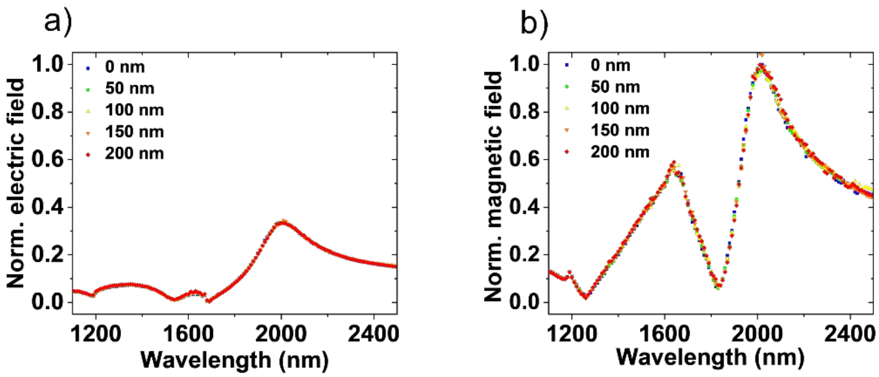

Figure S-5: Effect of residual SiO_2 layer on VO_2 metasurface *(a) Normalized electric field and (b) normalized magnetic field confined in the VO_2 nanodisks are modeled for various thicknesses of residual SiO_2 hard mask on top of the VO_2 nanoantennas. Both plots are normalized to the magnetic field strength of the VO_2 nanodisks with no residual SiO_2 layer on them.*

The details of the elemental composition of the sample after the VO_2 etch step is shown below. This was useful to help model the effect of the resulting nanostructure composition on the optical performance of the VO_2 metasurfaces measured in this work.

a)

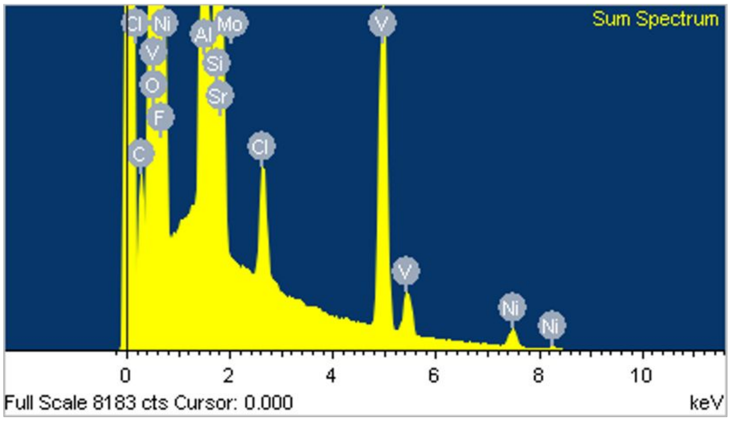

b)

Element	Weight%	Atomic%
C K	8.68	13.00
F K	26.03	24.62
Al K	7.35	4.90
Si K	10.84	6.94
Cl K	0.62	0.31
V K	2.35	0.83
Ni K	0.21	0.06
Sr K	0.00	0.00
Mo L	0.00	0.00
O	43.92	49.34
Totals	100.00	

Figure S-6: *(a). Energy dispersive x-ray spectrograph showing the residual elemental composition on a VO_2 metasurface sample after the last etch step. Carbon – from redeposited fluorocarbons and organics, Fluorine – from redeposited fluorocarbons and silicon fluoride compounds, Chlorine – from redeposited vanadium and aluminum chlorides, Aluminum – from sapphire handle wafer and alumina ceramic insulation in the reactor chamber, Vanadium – from nanoantenna material, Silicon – from fused quartz substrate, Oxygen – from nanoantenna material, substrates, and organics, Nickel – unknown. Molybdenum and Strontium are spurious and do not contribute to the overall elemental composition of the sample measured. (b). Table showing elemental composition on VO_2 metasurface sample area measured.*

Section 6: VO_2 Metasurface Spectral Characterization

The fabricated samples were mounted to the donut-shaped ceramic heater (described in the main text) and measured using a Perkin Elmer spectrometer with integrating sphere. Below are the results from the measurements before background and intensity corrections were made to normalize the data. This normalization allows spectral comparisons that take into account intensity losses in both the experimental set-up and in the fabrication residues on the nanoantennas.

The deviation of the data from the model is attributed to an imperfect fabrication pathway for making these VO_2 nanocylinder arrays. Fabrication defects are mostly due to redeposition occurring in both etch steps in the process. While ion-milling helps to reduce some of the residue, the metasurface electron micrograph and modulation performance suggests that more etch residue may need to be removed to make the fabricated metasurface perform better and closer to their predicted modeled outcomes. Wet methods such as buffered oxide etch and deionized water clean at elevated temperatures (50°C) were attempted to remove the etch residue but proved unsuccessful.

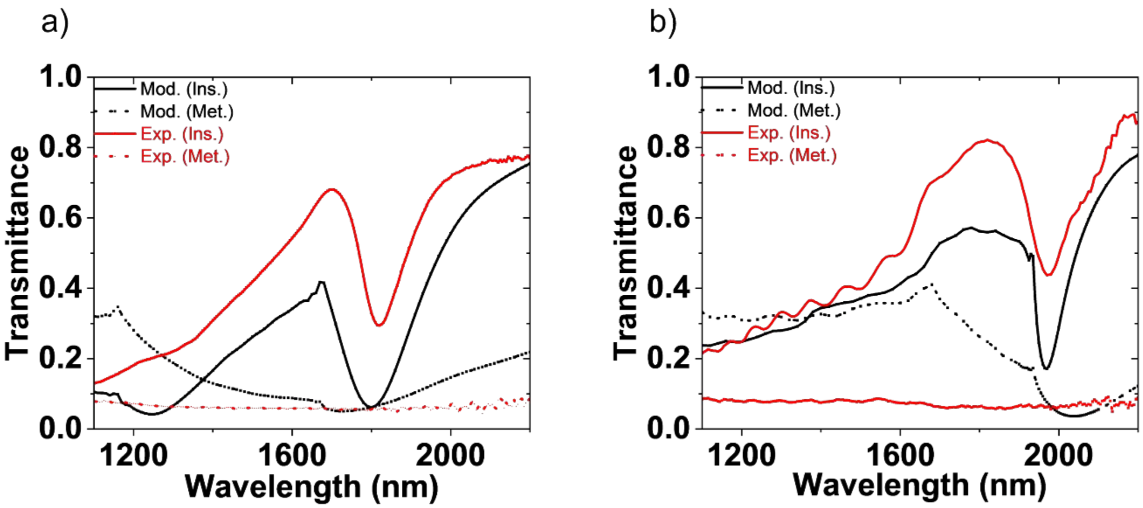

Figure S-7: Spectral metasurface characterization *(a) Transmittance vs. wavelength plot for both VO_2 metasurface insulating and metallic phases before encapsulating with PDMS. (b). Transmittance vs. wavelength plot for both VO_2 insulating and metallic phases after PDMS encapsulation. These plots compare the modeled spectra and the measured raw data from the spectrometer before the data is normalized for Figure 4.*

References

1. Falcone, F.; Lopetegi, T.; Laso, M. A. G.; Baena, J. D.; Bonache, J.; Beruete, M.; Marques, R.; Martín, F.; & Sorolla, M. Babinet principle applied to the design of metasurfaces and metamaterials. *Phys. Rev. Lett.* **2004** 93(19),. doi:10.1103/physrevlett.93.197401.
2. Shi, R.; Shen, N.; Wang, J.; Wang, W.; Amini, A.; Wang, N.; & Cheng, C. Recent advances in fabrication strategies, phase transition modulation, and advanced applications of vanadium dioxide. *Appl. Phys. Rev.* **2019** 6(1),. doi:10.1063/1.5087864.
3. Azad, S.; Gajula, D.; Sapkota, N.; Rao, A.; & Koley, G. Infrared Transmission Characteristics of Phase Transitioning VO2 on Various Substrates. *Micromachines* **2022** 13(5), 812. doi:10.3390/mi13050812.
4. Quartz single crystal, Z-cut, 10x10x0.5mm, 2sp. https://www.mtixtl.com/SO-Z-101005-S2.aspx.
5. Lee, S.; Ivanov, I. N.; Keum, J. K.; & Nyung Lee, H. Epitaxial stabilization and phase instability of VO 2 polymorphs OPEN. *Nat. Publ. Gr.* **2015** doi:10.1038/srep19621. doi:10.1038/srep19621.
6. Goodacre, D.; Blum, M.; & Buechner, C. Water adsorption on vanadium oxide thin films in ambient relative humidity. *J. Chem. Phys* **2020** 152 44715. doi:10.1063/1.5138959.
